# Apnea-Induced Cortical BOLD-fMRI and Peripheral Sympathoneural Firing Response Patterns of Awake Healthy Humans

**DOI:** 10.1371/journal.pone.0082525

**Published:** 2013-12-16

**Authors:** Derek S. Kimmerly, Beverley L. Morris, John S. Floras

**Affiliations:** 1 Clinical Cardiovascular Physiology Laboratory, University Health Network and Mount Sinai Hospital Division of Cardiology, Department of Medicine, University of Toronto, Toronto, Ontario, Canada; 2 School of Health and Human Performance, Faculty of Health Professions, Dalhousie University, Halifax, Nova Scotia, Canada; Université de Montréal, Canada

## Abstract

End-expiratory breath-holds (BH) and Mueller manoeuvres (MM) elicit large increases in muscle sympathetic nerve activity (MSNA). In 16 healthy humans (9♀, 35±4 years) we used functional magnetic resonance imaging with blood oxygen level-dependent (BOLD) contrast to determine the cortical network associated with such sympathoexcitation. We hypothesized that increases in MSNA evoked by these simulated apneas are accompanied by BOLD contrast changes in the insular cortex, thalamus and limbic cortex. A series of 150 whole-brain images were collected during 3 randomly performed 16-second end-expiratory BHs and MMs (-30 mmHg). The identical protocol was repeated separately with MSNA recorded from the fibular nerve. The time course of the sympathoexcitatory response to both breathing tasks were correlated with whole-brain BOLD signal changes. Brain sites demonstrating both positive (activation) and negative (deactivation) correlations with the MSNA time course were identified. Sympathetic burst incidence increased (p<0.001) from 29±6 (rest) to 49±6 (BH) and 47±6 bursts/100 heartbeats (MM). Increased neural activity (Z-scores) was identified in the right posterior and anterior insular cortices (3.74, 3.64), dorsal anterior cingulate (3.42), fastigial and dentate cerebellar nuclei (3.02, 3.34). Signal intensity decreased in the left posterior insula (3.28) and ventral anterior cingulate (3.01). Apnea both activates and inhibits elements of a cortical network involved in the generation of sympathetic outflow. These findings identify a neuroanatomical substrate to guide future investigations into central mechanisms contributing to disorders characterized by elevated basal MSNA and exaggerated sympathetic responses to simulated apneas such as sleep apnea and heart failure.

## Introduction

Obstructive and central apneas elicit entrained cyclical surges in efferent sympathetic vasoconstrictor discharge [1]–[3]. Relative to control participants, individuals with these two forms of sleep apnea exhibit increased central sympathetic outflow to skeletal muscle even during wakefulness [4], [5]. Importantly, across a broad range of cardiovascular conditions, augmented sympathetic nervous system activity has been linked to increased morbidity and mortality [6]–[9].

Although the key regulatory nuclei involved with the generation of sympathetic outflow are located within the brainstem, experimental evidence indicates that higher cortical regions also participate in its regulation [10]–[14]. However, in humans the central neural sources of sympathetic excitation in conditions such as sleep apnea have yet to be elucidated. Before we can determine whether aberrant central neural regulation occurs in pathological conditions characterized by exaggerated sympathetic outflow we must first determine the brain sites involved with its generation and regulation in healthy individuals. Functional magnetic resonance imaging (fMRI) provides a unique opportunity to address this knowledge gap.

To date, relatively few studies have integrated information obtained from fMRI-derived indices of brain neural activity with direct microneurographic recordings of muscle sympathetic nerve activity (MSNA) [15]–[18]. In previous experiments from our laboratory involving both healthy humans and heart failure patients, Mueller manoeuvres (MM, inspiratory efforts against resistance) and breath holds (BH) elicited intense sympathoexcitatory responses of similar magnitude but different time courses, in that sympathetic excitation persisted beyond the cessation of the Mueller manoeuvre [19]. This finding provides an opportunity to characterize the brain regions involved with the generation and/or modulation of sympathetic vasoconstrictor outflow *per se* at a time when any cortical neural patterns associated with the volitional motor effort, baroreceptor- and chemoreflex-mediated, emotional and sensory responses have dissipated [19].

The purpose of the current investigation was to use fMRI in awake healthy humans to determine the brain regions associated with the reflex increases in MSNA evoked by end-expiratory breath holds and Mueller manoeuvres. Based on the results of prior human neuroimaging studies [15], [18], [20]–[23], we hypothesized that the increases in MSNA would be accompanied by altered blood oxygen level-dependent contrast signaling within the insular cortex and discrete regions within the limbic system, such as the thalamus and anterior cingulate cortex.

## Methods

### Participants and Ethics Statement

Sixteen healthy volunteers [7♂ and 9♀, age 35±14 years (range 20–65 years), body mass index 23±4 kg/m^2^] provided written and verbal informed consent for the present investigation, which was approved by the Research Ethics Board of the University Health Network. All participants were unmedicated, normotensive non-smokers with no history of autonomic, sleep, cardiovascular or neurological disorders.

Two experimental sessions, the physiologic recording and the functional magnetic resonance imaging, were performed in a randomly assigned order on different days but at the same time of day. Participants were familiarized with the experimental procedures during a prior screening and orientation visit. Each individual completed a routine medical examination and MRI-readiness questionnaire to ensure safety within a high magnetic field environment. Participants arrived for each testing session approximately 3 hours after a light meal, which they were instructed to record in detail and replicate on the second study day, prior to the experimental test session. In addition, they were instructed to arrive well hydrated but to abstain from caffeinated, alcoholic and nicotine containing products for 24 hours before each test. Participants voided before instrumentation to minimize the effects of a distended bladder on sympathetic activity and arterial blood pressure [24].

### Apnea Protocol

A minimum 10 minutes of quiet supine rest and spontaneous breathing preceded the performance of three 16 second end-expiratory breath holds (BH, simulated central apneas) and Mueller manoeuvres (MM, simulated obstructive apneas). There were 30 seconds of rest that separated each apnea. The order of the 6 breathing tasks (3 BHs and 3 MMs) was randomly assigned between participants but kept consistent within each subject for the physiologic and fMRI test sessions. During the apnea protocol, participants wore a nose clip and breathed through a mouthpiece connected in series to a pressure transducer for the measurement of airway load pressure. After a stable baseline had been achieved, participants paused momentarily at end-expiration while a stopper with a small air leak was positioned into the end of the mouthpiece. Participants then generated and sustained -30 mmHg (∼41 cmH_2_O) of pressure for 16 seconds. A visual display of mouth pressure was provided continuously to help participants maintain the required level of negative pressure during the Mueller manoeuvres. All participants performed practice trials prior to data collection to familiarize themselves with these procedures and to ensure that the required level of negative pressure could be maintained for the entire 16 seconds. For the breath hold challenges, participants were instructed to hold their breath for 16 seconds following the end of a normal expiration.

### Laboratory data acquisition and analysis

Data were acquired in a quiet, temperature-controlled room with participants supine. Heart rate (HR) was calculated from successive R-R intervals recorded from Lead II of an electrocardiogram (ECG), respiratory movements were determined by a pneumobelt connected to a pressure transducer and continuous estimates of arterial blood pressure (ABP) were collected non-invasively using automated photoelectric plethysmography (Portapres® Model-2, Finapres Medical Systems BV, Amsterdam, The Netherlands) [25]. Portapres® blood pressure waveforms were corrected against non-invasive oscillometric determinations of brachial artery pressure in the opposite arm (standard adult cuff: 23–33 cm; Dinamap® Pro 100, Critikon, Tampa, FL, USA), which were acquired each minute throughout the experimental protocol. Multi-unit recordings of post-ganglionic muscle sympathetic nerve activity were obtained with a unipolar tungsten microelectrode (FHC, Bowdoinham, ME) inserted percutaneously into a fascicle of the right common fibular nerve [26]–[28].

Analogue signals for blood pressure, respiratory movements, mouth pressure (200 Hz-sampling), the ECG (1000 Hz), and the raw neurogram (10 kHz) were collected with an on-line data acquisition and analysis system (Spike 5, CED, Cambridge, UK). Pre-breathing task baseline data were averaged over a minimum 60 second period before the first breath hold or Mueller manoeuvre. Peak MSNA and HR increases during each of the six breathing challenges were determined, and the mean of these responses was used for statistical analysis. Only pulse-synchronous bursts of MSNA activity with characteristic rising and falling slopes, and amplitudes that were at least thrice that of the between-burst baseline fluctuations (≥3∶1 signal/noise), were included in the analysis. After an acceptable neural recording was obtained, the participant was instructed to maintain the leg in a relaxed position for the remainder of the study. Segments of the neural recording that showed evidence of mechanoreceptor activity caused by muscle tension were excluded from analysis. MSNA was analyzed for frequency (bursts per minute) and incidence (bursts per 100 heartbeats).

All physiologic data are expressed as means ± SD. Breath hold- and Mueller manoeuvre-mediated changes in all dependent variables were analyzed using a repeated-measures one-way ANOVA. Tukey's post hoc analysis was performed to estimate differences among means. Probability levels during multiple point-wise comparisons were corrected using Bonferonni's approach. Significant differences between breath hold and Mueller manoeuvre were analyzed using a paired t-test. Statistical analyses for HR, end-tidal carbon dioxide (ETCO_2_, see below) and MSNA data were performed using a computer-based software program (SAS). A critical significance level of p≤0.05 was set for all physiologic comparisons.

### fMRI Data Collection and analysis

All brain images were collected using an 8 channel phased array head coil while participants lay supine in a 3 Tesla whole body scanner (Signa, General Electric Healthcare, Milwaukee, WI, USA). Foam pads were positioned on either side of the head, under the elbows and knees to minimize head movement and participant discomfort during scanning. Gradient-echo echo-planar imaging-sensitive blood oxygen level-dependent (BOLD) contrast was used to identify cortical regions in which blood flow and/or metabolism changed (from baseline) during each of the apneas [29], [30]. BOLD fMRI signal changes have been shown to correlate with local field potentials and reflect input and intracortical processing [31], [32]. A continuous time series of 150 whole-brain image volumes were collected (time to repetition  = 2 s, time to echo  = 30 ms, flip angle  = 85°, field of view  = 240 mm). Each volume consisted of 32 interleaved contiguous axial slices (raw voxel size: 2.7×2.7×4.4 mm thick, no gap). Each functional scanning period was divided into an initial 30 second period of quiet rest (15 volumes) followed by 6 repetitions that consisted of a 16 second apnea (8 volumes) and a 30 second rest period (15 volumes). In addition, a high resolution three-dimensional T1-weighted anatomical image covering the same levels as the functional images (voxel size  = 0.9×0.9×3.0 mm thick) was collected, where T1-weighted refers to images based on a tissue-dependent recovery of the longitudinal component of net magnetization over time.

The methods and equipment described above for the apnea protocol were repeated during scanning. In addition, MRI-compatible goggles provided continuous visual feedback regarding the level of developed negative airway load pressure during the Mueller manoeuvres and headphones were used to provide instructions (e.g., “Begin Mueller manoeuvre”, etc.) and communicate with the participants during and between scanning periods. Continuous ECG and ETCO_2_ measures were obtained during the neuroimaging sessions using an MRI-compatible vital signs monitor (Veris, Medrad Inc., Warrendale, PA, USA). Baseline ETCO_2_ data were averaged over all complete respiratory cycles collected during the 30 second period before the first breathing challenge. In addition, the ETCO_2_ during the first breath following each breathing challenge was compared to baseline.

Whole cortical BOLD fMRI images were analyzed using statistical parametric mapping software (SPM8, Wellcome Department of Cognitive Neurology) [33]. Following removal of the first three image volumes to account for scanner saturation, all image volumes were corrected for slice acquisition timing and realigned to correct for motion-related artifacts using the INRIAlign toolbox [34]. Due to technical limitations and participant head size, we were unable to obtain complete BOLD fMRI data sets in all participants (n = 9) from the inferior segments of the cerebellum. As such, primary analysis was focused on regions within the cerebral cortex. However, fMRI data from the 12 participants with complete cerebellar scans have been included and presented together with the cortical results. The functional images were then spatially normalized into a fixed stereotactic space using the Montreal Neurological Institute (MNI) coordinate system such that image volumes for all participants were in the same three-dimensional space. To suppress noise and effects due to inter-subject differences in functional and cortical anatomy, these spatially normalized image volumes were Gaussian smoothed with a kernel set at 8 mm full width at half maximum. To minimize the influence of low frequency noise, all functional images were temporally smoothed using a high-pass filter with a 90 second time constant (i.e., ∼0.011 Hz). Finally, the smoothed images were detrended to account for global signal intensity changes (e.g., due to changes in CO_2_ and/or perfusion variations) using the Linear Model of the Global Signal (LMGS) extension for SPM8 [35] prior to statistical inference.

**Figure 1:**
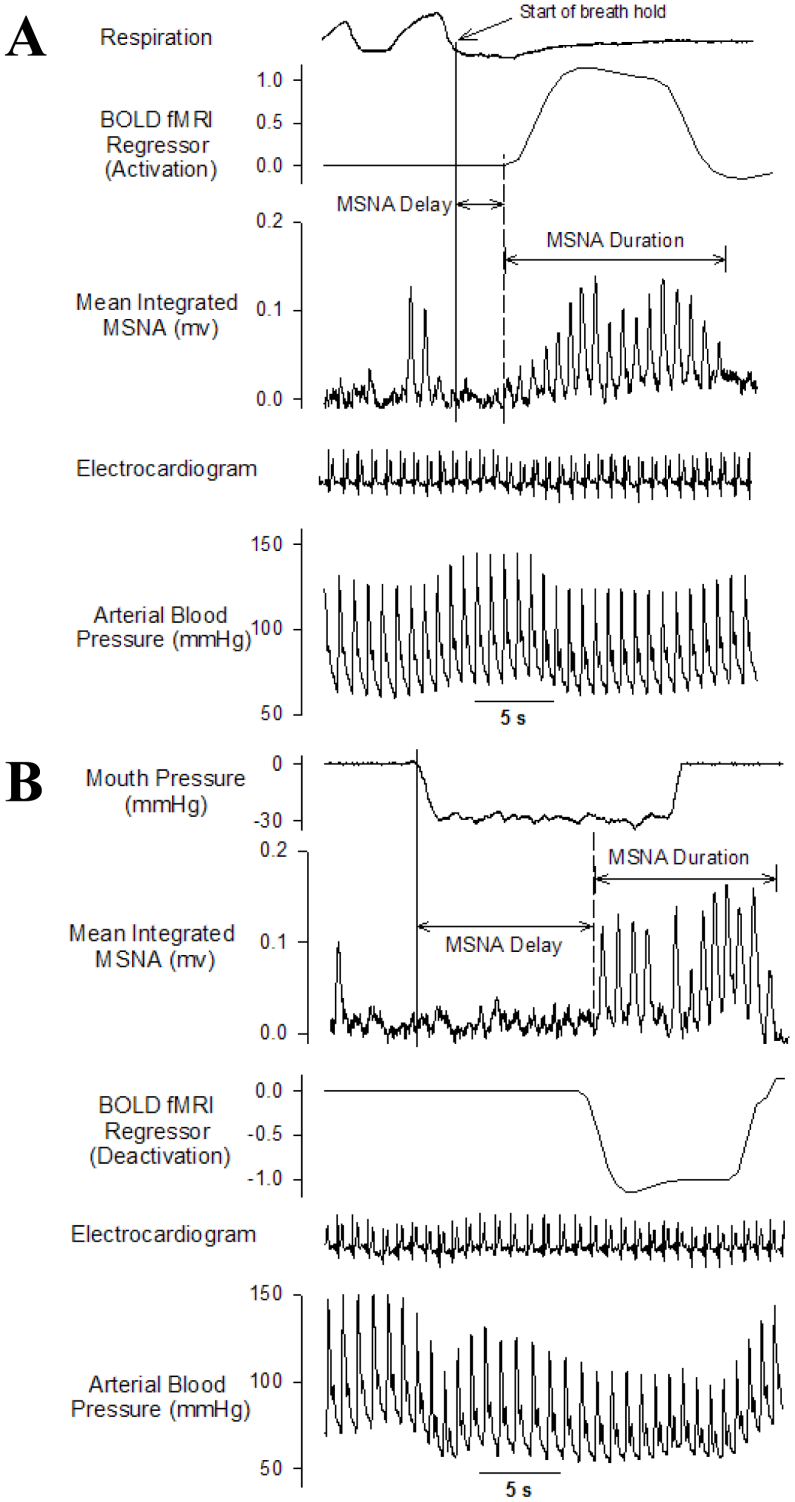
Example of the MSNA and BOLD-fMRI responses to an end-expiratory breath hold and Mueller manoeuvre. Representative data highlighting the changes in muscle sympathetic nerve activity (MSNA) mean voltage neurogram, arterial blood pressure, electrocardiogram and the blood oxygen level-dependent (BOLD) functional magnetic imaging (fMRI) regressor models during a 16 second end-expiratory breath-hold (A) and -30 mmHg Mueller manoeuvre (B). Note: the BOLD-fMRI regressor example during the breath-hold represents a model of neural activation (increased BOLD signal intensity) whereas the example for the Mueller manoeuvre depicts neural deactivation (i.e., a negative contrast was used on the activation regressor). Both positive (activations) and negative (deactivations) contrasts were examined for the sympathoexcitatory responses elicited by each of the two apneas. On average, the time delay between the start of the breathing task to the elicited MSNA response was longer for the Mueller manoeuvre resulting in elevated MSNA following the cessation of the apnea. However, the duration of the sympathoexcitatory response was similar between the two apneas.

Only the BOLD-fMRI data acquired during the intervals exhibiting sympathetic excitation for all 6 apneas were included for subsequent correlational analysis. This resulted in a temporal model of cortical neuronal activity patterns representative of the apnea-induced sympathetic response *per se*, while attempting to minimize cortical neural activity changes influenced by volitional, baroreceptor, chemical and/or sensory stimuli. For each participant, the average time course of the MSNA response elicited by the six breathing challenges was characterized and used to construct a model that was correlated with the BOLD fMRI time series data at each individual voxel in the brain. Specifically, the MSNA time course profile involved determination of the time delay (i.e., time in seconds from the start of each breathing task to the marked increase in MSNA) and the total duration of the MSNA response for each participant (Please see Fig. 1 for an example). Finally, to account for the hemodynamic lag associated with the BOLD response, the BOLD fMRI regressor began three image volumes (∼6 seconds) prior to the start of the sympathoexcitatory response elicited by each apnea. Based on these temporal parameters, the MSNA time series was correlated with the fMRI time series from each voxel. The activation regressor was modeled, and then both activation and deactivations were assessed using positive and negative contrasts, respectively. This resulted in subject-specific contrast images containing whole brain information related to both sites of increased and decreased BOLD signal during the sympathoexcitation induced by the breathing challenges. Significant voxels (p<0.001, uncorrected) were color coded for T-score and overlain onto a normalized high resolution anatomical image. A minimum cluster threshold of 10 voxels was included. All functional MRI data are represented by neurological convention (i.e., participant's left appears on the left).

## Results

### Physiological Responses to the Apneas

**Figure 2:**
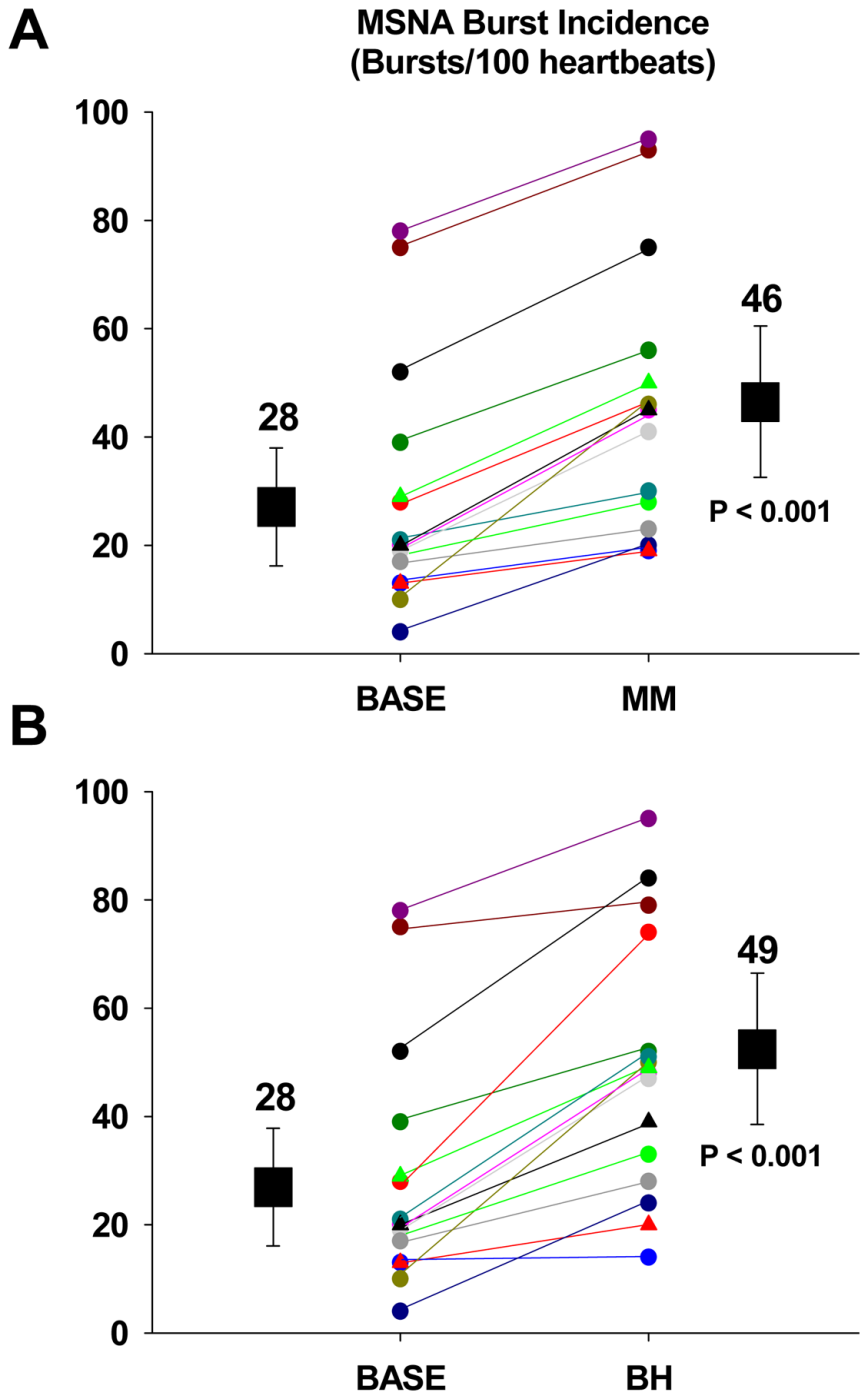
Mean MSNA response to the apneas. Individual and group mean (±SD) MSNA burst incidence data, at rest (BASE), during a 16 second MM (A) and BH (B). There were no statistical differences in the magnitude of the MSNA response between the 2 apneas (p = 0.68). MM, end-expiratory Mueller manoeuvre; BH, end-expiratory breath hold.

Figure 1 illustrates in one volunteer changes in MSNA, ABP, and HR during a 16 s end-expiratory breath hold and Mueller manoeuvre. Consistent with our previous observations [19], there was a longer (p<0.001) time delay between the initiation of the Mueller manoeuvre and the peak sympathoexcitatory response (13±6 seconds) than for the breath-hold (4±4 seconds). On average, the MSNA response elicited by the MM encompassed the final 3 seconds of the manoeuvre itself plus the first 5 seconds of recovery. However, the duration of the sympathoexcitation was similar between the two apneas (8±4 seconds and 10±4 seconds for the MM and BH, respectively). There was a greater (p = 0.02) peak HR response to the MM than the BH (81±15 beats/min vs. 70±12 beats/min, respectively). However, the increase in MSNA burst incidence from baseline (28±21 bursts/100 heartbeats) was similar (p = 0.68) for both the MM (Δ18±8 bursts/100 heartbeats) and BH (Δ21±12 bursts/100 heartbeats). Individual and mean MSNA burst incidence responses to the MM and BH are displayed in Figures 2A and B, respectively. The end tidal CO_2_ values from the expiration immediately following the BHs (40±7 mmHg) or the MMs (41±7 mmHg) were not different (p>0.18) from the baseline values obtained during the last minute prior to the start of the apnea protocol (40±5 mmHg).

### BOLD Signal Changes Associated with the Sympathoexcitatory Response to MM and BH

**Figure 3:**
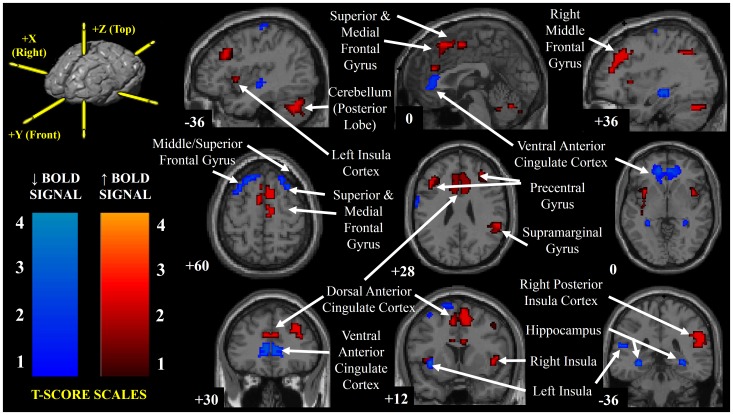
Brain regions that demonstrated significant BOLD signal changes associated with apnea-induced sympathoexcitation. Highlighting brain regions that demonstrated significant increases and decreases in BOLD fMRI signal intensity (coded for T-score using a hot or winter color scale, respectively) that correlated with the time course of the MSNA response elicited by the MM and BH. Montreal Neurological Institute slice position coordinates are displayed at the bottom left of each image (the reference coordinate system schematic is located in the top left pane).

**Figure 4:**
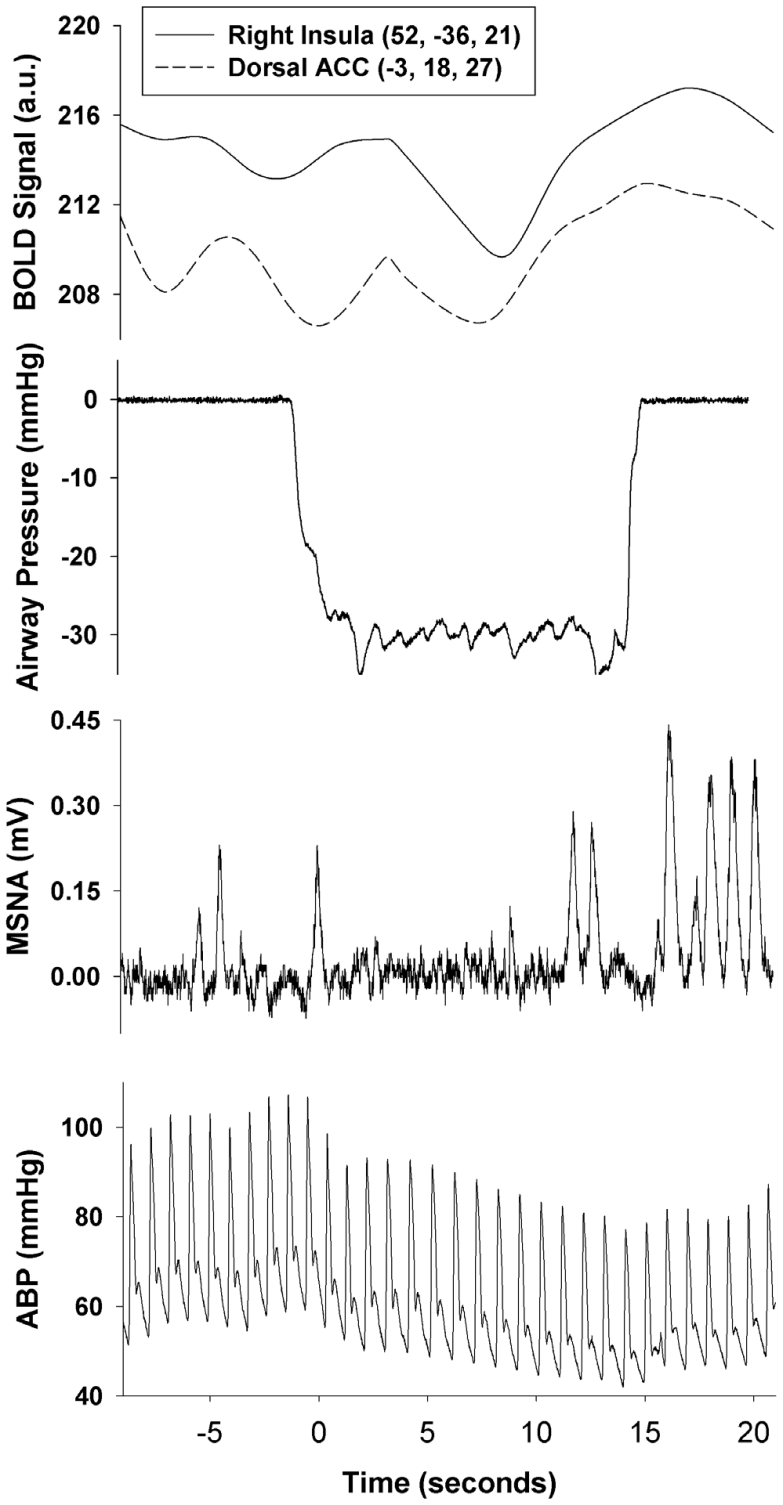
Example time course of brain regions that followed the MSNA temporal pattern during a Mueller Manoeuvre. Representative physiologic and BOLD fMRI responses to a Mueller Manoeuvre highlighting the inhibition of MSNA during the apnea followed by a brief period of sympathoexcitation following termination of the manoeuvre. The corresponding BOLD signal time course for the dorsal anterior cingulate cortex (−3, 18, 27) and right posterior insular cortex (51, −36, 21) have been included. The coordinates in parentheses represent the voxel with the greatest statistical value (Z-Score) within each cluster.

The MNI coordinates and statistical scores of brain regions that demonstrated significant increases and decreases in BOLD signal intensity are shown in Tables 1 and 2 and Figure 3. Regions of increased BOLD signal were observed in the right posterior insula, bilateral anterior insular cortices, dorsal anterior cingulate cortex, precentral gyrus [Brodmann Area (BA) 9], anterior and posterior lobes of the cerebellum, and widespread regions of the frontal cortex (Table 1, Fig. 3). Figure 4 displays the temporal response within the right posterior insular cortex and dorsal anterior cingulate cortex (please refer to Table 1 and Figure 3), which follows the time course of the MSNA signal during a Mueller Manoeuvre in a representative participant. Decreases in BOLD signal intensity that matched the time course of the MSNA signal during the breathing tasks were located within discrete regions of the bilateral precentral gyrus (BA 6), frontal cortex, ventral anterior cingulate cortex, left posterior insula and hippocampus (Table 2 and Fig. 3).

**Table 1:** MNI coordinates of brain regions that showed increased BOLD fMRI signal intensity during the sympathetic vasoconstrictor response elicited by the apneas.

Brain Region (Brodmann Area #)	MNI Coordinates (x,y,z)	Voxels (#)	Z-Score
Superior Frontal Gyrus (6)	6, 15, 54	85	3.95
	−6, 18, 57	79	3.80
Medial Frontal Gyrus (6)	9, 9, 51	105	3.31
	−6, 6, 51	27	3.88
Right Middle Frontal Gyrus (9)	33, 33, 39	42	3.68
	39, −3, 51	52	3.63
Precentral Gyrus (9)	36, 21, 36	42	3.39
	−39, 24, 36	10	3.60
Dorsal Anterior Cingulate (24,32,33)	−3, 33, 27	95	3.37
	9, 12, 27		2.91
	9, 24, 27		3.42
Right Posterior Insular Cortex (13)/Supramarginal Gyrus (40)	51, −15, 12	16	3.74
	54, −36, 33	179	3.17
Insular Cortex (13)	42, 9, 0	14 24	3.64 3.32
	−36, 12, 3		
Cerebellum (Anterior Lobe)	15, −54, −30	224	2.99
	−24, −51, −18		2.75
Cerebellum (Fastigial Nucleus)	3, −48, −21	13	2.71
	−3, −51, −27		3.02
Cerebellum (Posterior Lobe)	39, −69, −21	396	3.64
	−24, −72, −21		3.29
Cerebellum (Dentate Nucleus)	12, −66, −33	102	3.30
	−12, −69, −36	89	3.34

The x- (right/left), y- (anterior/posterior), and z- (superior/inferior) values represent the MNI coordinates (in millimeters from the anterior commissure) for the voxel with the greatest statistical value (Z-Score) within each cluster.

*Definition of Abbreviations*: BOLD  =  blood oxygen level-dependent; fMRI  =  functional magnetic resonance imaging; MNI  =  Montreal Neurological Institute.

**Table 2:** MNI coordinates of brain regions that showed decreased BOLD fMRI signal intensity during the sympathetic vasoconstrictor response elicited by the apneas.

Anatomical Region (Brodmann's Area #)	MNI Coordinates (x,y,z)	# Voxels	Z-Score
Precentral Gyrus (6)	27, −9, 72	69	2.97
	−27, −9, 69	10	3.17
Middle/Superior Frontal Gyrus (6)	−9, 12, 69	16	2.78
	27, 21, 63	12	2.84
	−30, 21, 60	31	3.19
Ventral Anterior Cingulate Cortex (24,25)	3, 36, 3	63	3.01
	−3, 39, 3	88	2.81
Left Posterior Insular Cortex (13)	−57, −33, 18	14	3.28
	−39, −24, −3	14	2.70
Hippocampus	33, −33, −12	25	3.26
	−30, −30, −9	11	3.24

The x- (right/left), y- (anterior/posterior), and z- (superior/inferior) values represent the MNI coordinates (in millimeters from the anterior commissure) for the voxel with the greatest statistical value (Z-Score) within each cluster.

*Definition of Abbreviations*: BH  =  end-expiratory breath hold;

BOLD  =  blood oxygen level-dependent; fMRI  =  functional magnetic resonance imaging; MM =  end-expiratory Mueller manoeuvre; MNI  =  Montreal Neurological Institute.

## Discussion

The aim of the current investigation in awake healthy humans was to identify, using BOLD fMRI time series images, the autonomic cortical network associated with apnea-induced muscle sympathetic nerve activity. Brain regions engaged by the MM and BH included the insular cortex, anterior cingulate cortex and cerebellum. In the present study there was concordance between the brain regions highlighted and those previously reported to be involved with the sympathetic response elicited by the non-volitional stresses of lower body negative pressure [15], [20], [21] and inspiratory capacity apnea [18]. Taken together, these findings provide a foundation from which, in future studies, the central neural structures involved with disease states associated with pathological elevations in resting sympathetic nervous system activity and disordered breathing may be identified, explored with finer resolution, and perhaps targeted therapeutically.

Although they do not replicate precisely the complex neural and chemical events triggered by obstructive and central apnea during sleep, the performance of such interventions, which are amenable to standardization, by awake volunteers has yielded novel insight into the magnitude and time courses of the hemodynamic and neural responses elicited by these stimuli in health and disease [2], [19], [36]–[38]. Consequently, MM and BH have been adopted by several groups investigating the acute cardiovascular and autonomic responses of sleep apnea. Consistent with previous reports [19], [36], the magnitude of the peak sympathoexcitatory response was similar for the end-expiratory Mueller manoeuvres and breath holds. As anticipated, however, and as illustrated in Figure 1, the initial MSNA responses and the time courses of subsequent sympathoexcitation differed. We therefore synchronized the BOLD activity data to the specific timing of BH- and MM-induced muscle sympathoexcitation.

With BH, this sympathoexcitation occurred sooner than with the MM. The increase in MSNA elicited by BH is mediated primarily by peripheral chemoreflex activation [36], [39] and by loss of normal sympathoinhibition exerted during breathing by afferent input from pulmonary stretch receptors [40], [41]. MSNA continues to rise during the BH but returns to baseline once spontaneous breathing resumes (Figure 1, A). By contrast, the MM evokes a triphasic response (Figure 1, B). The large intra-thoracic pressure generated abruptly at the start of the MM increases aortic transmural pressure, thereby activating aortic arch baroreceptors and resulting in an initial period of reflex sympathoinhibition. Subsequent unloading of carotid arterial and possibly low-pressure baroreceptors, and absence of inhibitory pulmonary stretch receptor input, causes reflexively a recovery of MSNA to baseline. Finally, once carotid chemoreceptors become stimulated by hypoxia, there is a sustained elevation of MSNA above baseline levels. Such peripheral sympathetic activation persists beyond cessation of the inspiratory effort [42], [43]. Although it has been suggested that this can be enhanced if medullary chemoreceptors are stimulated by hypercapnia [36], the absence of any change in the end-tidal CO_2_ values following the MM and BH (likely because both were performed at end-expiration and were of relatively short duration) argues against significant engagement of this population of receptors by the present protocol.

Because of the relatively large between-subject variations in the time delay (BH, 1–13 seconds; MM, 1–26 seconds) and duration (BH, 5–16 seconds; MM, 5–15 seconds) of the sympathoexcitatory responses, for each participant, the temporal BOLD regression model was based on their specific MSNA time delay and duration of sympathoexcitation elicited by the BHs and MMs, respectively. In comparison, the within-subject variations in the MSNA time delay (BH, 0–5 seconds; MM, 0–6 seconds) and duration (BH, 1–5 seconds; MM, 0–6 seconds) were relatively consistent. Therefore, we applied a statistical paradigm, which ensures that between-subject differences in the temporal MSNA response are accounted for (rather than using a group averaged analytical approach, in which the group mean time profile of the MSNA response is correlated with the BOLD fMRI time series for each participant) [18]. To overcome the difficulty encountered in displaying a group-averaged time course of the BOLD signal in brain regions that increased or decreased activity during the apneas (due to the large between-participant temporal variation), we constructed traditional statistical parametric maps identifying regions displaying significant BOLD signal increases and decreases associated with the MSNA responses common to both apneas.

Two brain regions that demonstrated increased BOLD signal intensity matching the time course of the apneic MSNA responses were the right insular cortex and the dorsal portion of the anterior cingulate cortex (ACC). Both have been implicated as important cortical structures involved in the regulation of cardiovascular function. Direct electrical stimulation of the human right insula [44] and clinical neuroimaging investigations focused on lesions of the ACC [45] have highlighted the involvement of these cortical structures with sympathetic neural control. Increased signal fMRI intensity of both brain regions also has been associated with the excitatory MSNA response to non-volitional baroreceptor unloading using lower body negative pressure [15], [20], [21]. When late onset increases in BOLD signal within the anterior cingulate cortex were observed during an inspiratory capacity apnea, these were attributed to the voluntary suppression of breathing rather than to sympathoexcitation [18]. Because the anterior insula and ACC have been implicated in processing the unpleasantness of perceived dyspnea [46] and the relief of dyspnea [47], respectively, even though BOLD signal increases in right insula and dorsal ACC in the present experiments were concordant with the time course of the excitatory MSNA response, these structures may be linked also with sensory and motor components of the MM and BH.

The role of other brain regions that displayed increased neural activation during the sympathoexcitatory response to the apneas, including the supramarginal gyrus, precentral gyrus, superior, middle and medial frontal gyri, are less well understood with respect to autonomic cardiovascular function. Activation of these regions has previously been observed during respiratory challenges including inspiratory loading [48] and Valsalva's manoeuvre [12], [49]. In addition, neural activity of the middle frontal and precentral gyrus increased during other sympathoexcitatory stimuli such as moderate lower body suction, handgrip exercise and mental stress [15], [50]. The current findings suggest that these cortical areas may be involved with the disinhibition, excitation or feedback regulation of central sympathetic outflow.

It has long been recognized that the cerebellum plays a significant role in the regulation of autonomic cardiovascular function [51]. For example, stimulation of the fastigial region of the cerebellum increases heart rate, blood pressure and vasopressin release [52], [53], and in conscious humans, activation of deep cerebellar nuclei has been observed in conjunction with sustained sympathoexcitation induced by an inspiratory capacity [18]. Since cerebellar cortex and deep nuclei gray matter loss [54], [55] and aberrant fMRI signal changes associated with inappropriate cardiovascular responses elicited by expiratory [56] and inspiratory loading [49] have been observed in obstructive sleep apnea patients (compared to control participants), future studies of the broader central neural network involved in the abnormalities of circulatory regulation in sleep apnea should investigate as well discrete regions of the cerebellum.

Several loci, including the ventral portion of the ACC and the left posterior insular cortex, exhibited decreased fMRI BOLD signal intensity during the periods of apnea-evoked elevated MSNA. These structures may have sympathoinhibitory functions during basal conditions. Their deactivation may be required for reflex-mediated sympathoexcitation to fully emerge. A similar pattern of decreased BOLD signal intensity within these two brain areas has been reported to accompany also the increased sympathetic vasoconstrictor response elicited by baroreceptor unloading [15], [20], [21]. Electrical stimulation of the left insular cortex in epileptic patients is associated with predominantly depressor responses, an observation also consistent with a sympathoinhibitory role. In addition, stroke damage to the left insula has been linked with increased basal sympathetic tone [57]. The subdivisions of the ventral ACC identified in the present study overlap with the ventral medial prefrontal cortex (MPFC), which has also been considered a cardiovascular depressor region [58]. Stimulation of the rodent MPFC reduced the discharge of sympathoexcitatory neurons within the rostral ventrolateral medulla [58].

Other cortical areas that demonstrated a similar pattern of fMRI signal change included discrete regions of the frontal cortex including the precentral gyrus, and middle/superior frontal gyrus, which encroaches upon parts of the premotor and supplementary motor areas. The main role attributed to the prefrontal cortex concerns executive function. The exact role of this brain area related to autonomic control is unclear but activation within a similar region has previously been linked with the processing of baroreceptor stimulation in humans [59]. Both the premotor and supplementary motor areas are involved with motor/volitional control including the muscles involved with breathing [60]. Therefore, in addition to a potential sympathoinhibitory role, the decreased BOLD signal observed within these areas may be the consequence of a reduced neural drive to the respiratory muscles during the periods of apnea.

Because high-frequency radio pulses emitted during the fMRI scanning sequences saturate the microneurographic recording, it is not possible to collect simultaneously both signals during the same session. We elected to acquire BOLD signal intensity images and MSNA recordings on two separate days but according to a stringent protocol designed to minimize day-to-day variability of our signals. Beforehand, each participant engaged in apnea training sessions and on both study days, participants followed a standardized protocol prior to each experimental session to ensure that diurnal variation, hydration status, daily physical activity, sleep duration, nutritional content and consumption time of the pre-test meal were all controlled for. A recent investigation, in which a protocol of 4 second quiescent periods between 4 second BOLD fMRI scanning periods was applied to capture intermittent recordings of a stable microneurographic signal, provides encouragement that this technical limitation might be overcome in the future [17].

### Limitations

Limitations to the current study include the need to record MSNA and BOLD fMRI data on separate experimental days. The corresponding assumption is that the magnitude and timing of the evoked MSNA response to the apneas are consistent over repeated sessions. Although the MSNA response to head-up tilt [61], lower body negative pressure (LBNP) and a cold pressor test [62] have been shown to be reproducible over repeated days we are unaware of similar findings for the sympathoexcitatory response to apneic challenges. Nonetheless, we were able to identify regions of increased and decreased BOLD signal in brain areas previously reported to be involved with the control of muscle sympathetic outflow during non-volitional LBNP [15], [20], [21], handgrip exercise [22] and inspiratory capacity apnea [18].

We acknowledge that the lack of increase in ETCO_2_ observed post-BH and MM may have been caused by a relatively large inspiration at the end of the apnea due to a corresponding increase in respiratory drive, resulting in a larger tidal volume and a dilution of alveolar PCO_2_, and that without continuous recordings of arterial blood pressure and blood gases (O_2_ and CO_2_) we cannot precisely isolate the relative contributions of baroreceptor, peripheral and central chemoreceptor sensory input on the cortical BOLD responses.

We have attempted to minimize the confounding influence that apnea-induced changes in blood gases and intracranial pressure may have on the BOLD signal. However, although the LMGS method was used, it is still possible that some of the observed BOLD signal changes may originate from non-neuronal factors. Furthermore, we did not observe significant BOLD signal changes within key regulatory regions within the brainstem involved with the generation of sympathetic vasoconstrictor outflow (e.g., RVLM). Factors that may have enhanced our ability to detect discrete brainstem autonomic nuclei include further minimization of participant head motion during the apneic challenges, and the inclusion of a second dedicated BOLD fMRI scanning session focused only on the brainstem. It is important to emphasize that the purpose of the present study was not to map cortical autonomic networks associated specifically with baroreceptor or chemoreceptor influences but to identify the brain regions involved with the generation or regulation of sympathetic vasoconstrictor outflow in response to these two apneic manoeuvres. Consequently, for each breathing challenge, fMRI image analysis focused on integrating data acquired over the time intervals when MSNA was highest and the potential influences of baroreflex and volitional stimuli were least. Identification of brain regions involved specifically with the detection and integration of these three afferent signals merits future investigation.

### Perspective and Conclusion

This study establishes the functional organization of the cortical brain network associated in awake healthy individuals with the sympathoexcitatory responses elicited by Mueller manoeuvres (as simulated obstructive apnea) and breath holds (as simulated central apneas). Importantly, a key strength of this investigation was its focus on healthy young individuals, in whom BOLD signal responses are unlikely to be affected unpredictably by age or the presence of co-morbidities. Specific brain regions that demonstrated increased BOLD signal that correlated with the time course profile of the MSNA signal included the right insular cortex, dorsal anterior cingulate cortex, cerebellum, and widespread areas of the fronto-parietal cortex. Conversely, brain areas that showed decreased fMRI signal during periods of augmented sympathetic vasoconstrictor outflow evoked by the apneas included the left insular cortex, ventral anterior cingulate cortex, precentral gyrus and hippocampus. It is plausible that these brain regions may be important for inhibiting sympathetic outflow in the rested state and that the removal of this sympathoinhibitory function during autonomic challenges is necessary to elicit the increases in MSNA observed during the apneas.

The current findings provide a neuroanatomical substrate that may be utilized to help elucidate potential regions of central autonomic dysfunction in patient populations characterized by exaggerated basal and apnea-induced increases in MSNA such as heart failure and sleep apnea [19]. Furthermore, this information may guide novel therapies (i.e., pharmacologic interventions and/or cortical excitation/inhibition therapy) directed specifically at the brain regions involved with the increased cardiovascular risk that excessive sympathetic activation poses for such individuals. However, it should be appreciated that although this cortical network might be representative of a manipulation that simulates airway obstruction, it may not replicate exactly the signal changes that occur in disease states characterized by obstructive and/or central sleep apnea. As such, future investigations should be conducted that compare the brain neural response patterns evoked by simulated versus spontaneously occurring central and obstructive apneas.
